# Vital Signs: Melanoma Incidence and Mortality Trends and Projections — United States, 1982–2030

**Published:** 2015-06-05

**Authors:** Gery P. Guy, Cheryll C. Thomas, Trevor Thompson, Meg Watson, Greta M. Massetti, Lisa C. Richardson

**Affiliations:** 1 Division of Cancer Prevention and Control, CDC

## Abstract

**Background:**

Melanoma incidence rates have continued to increase in the United States, and risk behaviors remain high. Melanoma is responsible for the most skin cancer deaths, with about 9,000 persons dying from it each year.

**Methods:**

CDC analyzed current (2011) melanoma incidence and mortality data, and projected melanoma incidence, mortality, and the cost of treating newly diagnosed melanomas through 2030. Finally, CDC estimated the potential melanoma cases and costs averted through 2030 if a comprehensive skin cancer prevention program was implemented in the United States.

**Results:**

In 2011, the melanoma incidence rate was 19.7 per 100,000, and the death rate was 2.7 per 100,000. Incidence rates are projected to increase for white males and females through 2019. Death rates are projected to remain stable. The annual cost of treating newly diagnosed melanomas was estimated to increase from $457 million in 2011 to $1.6 billion in 2030. Implementation of a comprehensive skin cancer prevention program was estimated to avert 230,000 melanoma cases and $2.7 billion in initial year treatment costs from 2020 through 2030.

**Conclusions:**

If additional prevention efforts are not undertaken, the number of melanoma cases is projected to increase over the next 15 years, with accompanying increases in health care costs. Much of this morbidity, mortality, and health care cost can be prevented.

**Implications for Public Health Practice:**

Substantial reductions in melanoma incidence, mortality, and cost can be achieved if evidence-based comprehensive interventions that reduce ultraviolet (UV) radiation exposure and increase sun protection are fully implemented and sustained.

## Introduction

Skin cancer is the most common form of cancer in the United States, and melanoma is responsible for the most skin cancer deaths with over 9,000 each year. An individual dying from melanoma loses an average of 20.4 years of potential life (1). Total melanoma treatment costs are about $3.3 billion annually in the United States (2). Melanoma is the fifth most common cancer for men, and is the seventh most common cancer for women. More than 90% of melanoma cases in the United States are attributed to skin cell damage from ultraviolet (UV) radiation exposure (3,4).

Sun-protective behaviors (e.g., using sunscreen, wearing sun-protective clothing, and seeking shade) can reduce harmful exposure to UV. Sunburns are a significant risk factor for melanoma (5,6). Nearly 40% of persons in the United States report sunburn each year (7), indicating that many are not adequately protecting their skin from damaging UV that can cause melanoma. *The Guide to Community Preventive Services* (Community Guide) (http://www.thecommunityguide.org/news/2014/skin-cancer.html) recommends multicomponent community-wide programs and educational, environmental, and policy interventions based on evidence that they increase UV protective behaviors, decrease skin damage that can develop into melanoma, and reduce health care spending (8,9). Community-level interventions to reduce sun exposure include providing sunscreen and shade, increasing the availability of protective clothing and hats, and scheduling activities before or after midday hours.

This report presents current melanoma incidence and death rates for 2011, projections of melanoma incidence rates and cases, mortality rates, and treatment costs through 2030, and describes the potential impact of a comprehensive skin cancer prevention program in the United States.

***Key Points***

- Melanoma incidence rates have doubled from 1982 to 2011.
- In 2011, in the United States, there were 65,647 cases of melanoma and 9,128 deaths.
- The annual cost of treating newly diagnosed melanomas is projected to triple by 2030.
- Melanoma can be prevented by reducing ultraviolet radiation exposure from sunbathing and indoor tanning and increasing the use of sun protection.
- A comprehensive national skin cancer prevention program could avert 230,000 melanoma cases and $2.7 billion in initial year treatment costs from 2020 to 2030.
- Additional information available at http://www.cdc.gov/vitalsigns.

## Methods

United States Cancer Statistics (USCS) (http://www.cdc.gov/uscs) provide official federal cancer incidence statistics in each state, using data from the National Program of Cancer Registries and the Surveillance, Epidemiology, and End Results (SEER) program. Forty-nine states and the District of Columbia (DC) met USCS publication criteria for 2011, representing 99.1% of the U.S. population. Incident melanomas of the skin were coded according to the *International Classification of Disease for Oncology, Third Edition*.

Cancer mortality statistics are based on all death certificates filed in the 50 states and DC, representing 100% of the U.S. population and provided by CDC’s National Center for Health Statistics. All reported deaths with melanoma of the skin identified as the underlying cause of death according to the *International Classification of Diseases, 10th Revision* were included.

Incidence and death rates for 2011 are presented per 100,000 persons and are age-standardized to the 2000 U.S. standard population. Population estimates produced by the U.S. Census Bureau were obtained from the SEER program (http://www.seer.cancer.gov/popdata). Corresponding 95% confidence intervals were calculated using the Tiwari method (10).

Incidence count and rate projections for whites are based on SEER data from 1982 to 2011, representing approximately 10% of the U.S. population (http://www.seer.cancer.gov). Death count and rate projections for blacks and whites are based on mortality data from 1982 to 2011. Population projections were obtained from the U.S. Census Bureau (http://www.census.gov/population/projections/data/national/2012.html). Age-period-cohort regression models were analyzed using statistical software, with assumptions that offset exponential increases or decreases in rates and that gradually reduced current trends over time (11). Projections were based on either long-term trend data or the most recent 10-year period data, depending on whether there was a statistically significant curvature in the trend over time. Predicted cancer incidence and death counts for the entire U.S. population were estimated by applying the age-specific rates to U.S. population projections.

To estimate the cost of melanoma treatment in the initial year of diagnosis, age- and sex-specific treatment costs were used (12). Cost estimates were adjusted using the per capita projected increase in national health expenditures through 2023 (http://www.cms.gov/Research-Statistics-Data-and-Systems/Statistics-Trends-and-Reports/NationalHealthExpendData/index.html). The annual rate of growth from 2024 through 2030 was calculated using the average increase in the 3 preceding years. Adjusted per capita treatment costs were multiplied by the projected number of new melanoma cases each year through 2030.

To determine the effectiveness of a comprehensive skin cancer prevention program in the United States, it is assumed that the observed reduction in melanoma incidence attributed to SunSmart (8), a multicomponent community-wide sun protection program in Australia, can be reproduced by a nationwide program in the United States. Similar to previous studies (9), the lag period between program implementation and reduced melanoma incidence was set at 5 years.

## Results

In 2011, a total of 65,647 invasive melanomas of the skin were reported in the United States (Table). The overall age-adjusted melanoma incidence rate was 19.7 per 100,000. Melanoma incidence rates increased with age, and were highest among non-Hispanic whites (24.6). Among persons aged 15–49 years, higher rates were observed among women, whereas among those aged ≥50 years, higher rates were observed among men.

In 2011, a total of 9,128 melanoma deaths occurred in the United States. The overall age-adjusted melanoma death rate was 2.7 per 100,000, with a higher death rate among non-Hispanic whites (3.4). Melanoma death rates increased with age and were higher among men (4.0) than among women (1.7).

**FIGURE 1:**
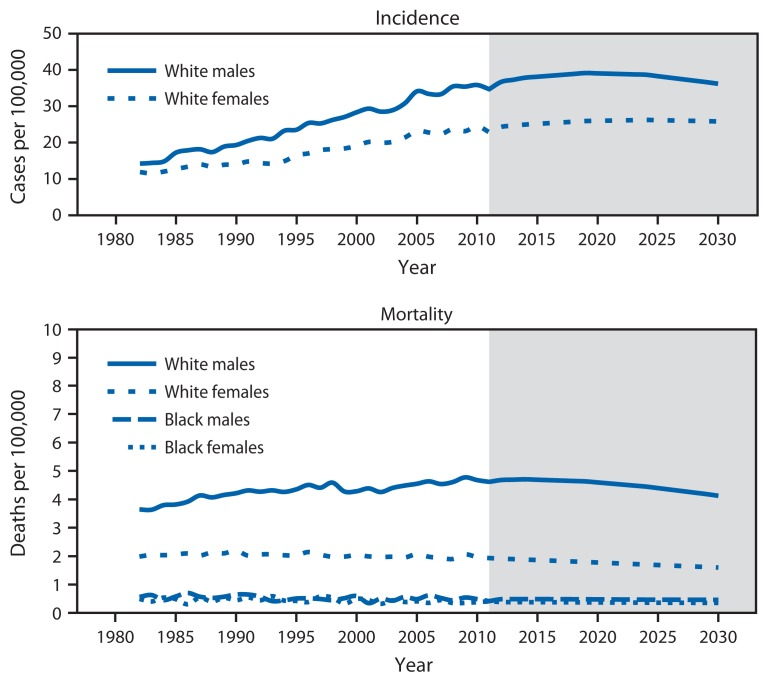
Observed and projected age-adjusted melanoma incidence and mortality rates, by sex and race — United States, 1982–2030* **Sources:** Melanoma incidence data are from the Surveillance, Epidemiology, and End Results program for the period 1982–2011. Mortality data are provided by CDC’s National Center for Health Statistics for the period 1982–2011. * Age-period-cohort regression models were used to project melanoma incidence and mortality rates through 2030.

**FIGURE 2:**
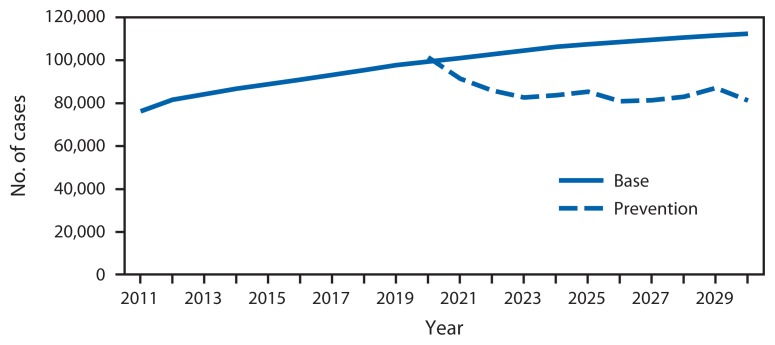
Annual observed and projected number of new melanoma cases among whites — United States, 2011–2030

From 1982 to 2011, melanoma incidence rates increased while mortality rates remained constant (Figure 1). Melanoma incidence rates doubled from 1982 to 2011. In the absence of new interventions, 112,000 new melanoma cases are projected in 2030 (Figure 2). A comprehensive skin cancer prevention program is estimated to prevent 20% of melanoma cases from 2020 to 2030, corresponding to an average of 21,000 melanoma cases averted each year (a total of 230,000 cases from 2020 to 2030).

**FIGURE 3:**
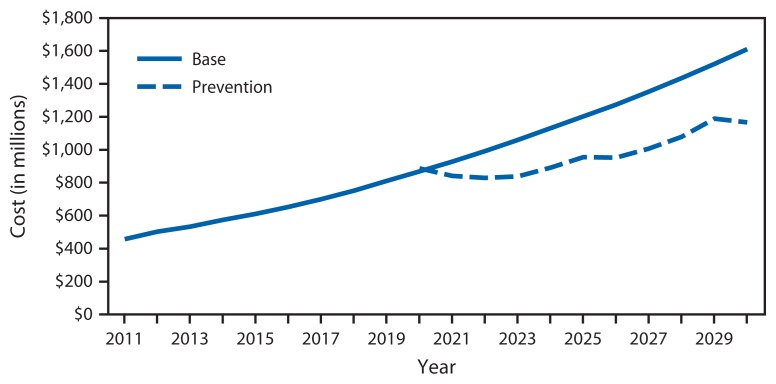
Annual observed and projected cost of treating new melanoma cases among whites — United States, 2011–2030

In the absence of new interventions, the annual cost of treating newly diagnosed melanoma cases is estimated to increase by 252.4% from 2011 to 2030 (from $457 million to $1.6 billion) (Figure 3). A comprehensive skin cancer prevention program is estimated to result in an average annual reduction in spending of $250 million on newly diagnosed melanoma cases, and a total of $2.7 billion during 2020–2030.

## Conclusions and Comment

The health and economic burden of melanoma is substantial and without additional efforts is projected to increase through 2030. Without new interventions, the annual cost of treating newly diagnosed melanomas is projected to increase threefold from 2011 to 2030. Prevention strategies include reducing UV exposure from sunbathing and indoor tanning, and increasing the use of sun protection (13). A comprehensive skin cancer prevention program was estimated to avert 230,000 melanoma cases and $2.7 billion in initial year treatment costs from 2020 through 2030.

The estimated impact of a comprehensive skin cancer prevention program on the number of melanoma cases and treatment costs averted was based on findings from SunSmart, an Australian skin cancer prevention program (8). SunSmart is a multicomponent, community-wide intervention designed to raise awareness, change personal behaviors, and influence institutional policy and practices. SunSmart activities include mass media campaigns; programs with schools, workplaces, and sports programs; health care provider education; resource development and dissemination; and building capacity for skin cancer prevention at the community level. SunSmart was estimated to prevent more than 9,000 melanomas, avert over 1,000 deaths, and save 22,000 life-years in the state of Victoria during 1988–2003. SunSmart has also been shown to save $2.30 for every $1 invested.

The Affordable Care Act is reducing financial barriers to preventive services by requiring many plans to cover clinical preventive services rated A or B by the U.S. Preventive Services Task Force (USPSTF) without patient cost sharing. Behavioral counseling is now provided with no cost-sharing to counsel individuals aged 10–24 years with fair skin about minimizing their exposure to UV radiation to reduce risk for skin cancer (14). USPSTF has stated that current evidence is insufficient to recommend skin cancer screening; an updated recommendation is in progress (http://www.uspreventiveservicestaskforce.org/Page/Topic/recommendation-summary/skin-cancer-screening?ds=1&s=).

Although rates of melanoma are highest among whites, persons who identify as non-white are also at risk for melanoma. Lack of awareness might result in underestimating risk. Blacks are more likely to report experiencing frequent sunburns (7) and less likely to engage in certain protective behaviors, particularly sunscreen use, compared to other racial/ethnic groups (15). Melanoma survival is poorest among black populations who develop it in non–sun-exposed skin, possibly because of later diagnosis, lower perceived risk among patients and physicians, and a higher proportion of certain types of melanoma with poorer survival (16–18).

Previous research suggests that melanoma trends reflect increases in cumulative exposure to UV and increases in skin cancer awareness and early detection. Despite increases in melanoma incidence, decreases in melanoma mortality among persons aged <65 years have been observed, likely reflecting earlier detection and improved treatment. Meanwhile, increasing melanoma mortality rates among persons aged ≥65 years and increasing incidence for both thin and thick lesions, along with the substantial contribution of thin lesions at diagnosis to melanoma mortality (about 30%), suggest that cumulative overexposure to UV radiation plays a substantial role (19).

This report found that among persons aged 15–49 years higher melanoma incidence rates were observed among women, whereas among those aged ≥50 years, higher rates were observed among men. Higher rates among young females compared with young males might be attributable, in part, to the widespread use of indoor tanning among females, which is associated with an increased risk for melanoma (20). Nearly one-third of non-Hispanic white women aged 16–25 years engage in indoor tanning each year (21). Meanwhile, higher melanoma rates among older non-Hispanic white men may be attributable, in part, to lower rates of sun protection and more time spent outdoors throughout life compared with women (15,22). Additionally, men are less likely to use sunscreen compared to women (15); thus, clothing and wide-brimmed hats might be particularly effective sun protection options for males, as well as increasing the use of sunscreen.

The findings in this report are subject to at least six limitations. First, delays in melanoma reporting might result in an underestimate of cases; reporting delays are more common for cancers such as melanoma that are often diagnosed and treated in nonhospital settings such as physicians’ offices. Second, incidence projections are based on data that represent approximately 10% of the US population and have a lower percentage of whites. Third, accurate confidence intervals are not available for the incidence and death projections. Fourth, the impact of a skin cancer prevention program is based on the assumption that a reduction in incidence could be achieved in 5 years. Fifth, the impact of a prevention program is extrapolated from a state in Australia to all of the United States, which has a different underlying population and health care system. Finally, cost estimates only include health care costs incurred in the initial year after diagnosis.

Although the burden of melanoma is increasing, it is estimated that a substantial number of new melanoma cases could be prevented by following effective prevention strategies to reduce sun exposure, facilitate sun protection, prevent sunburn, and reduce indoor tanning. A comprehensive skin cancer prevention program addressing these skin cancer risk factors can help slow the growth in melanoma incidence and reduce melanoma treatment costs.

## Figures and Tables

**TABLE:** Number and rate of new melanoma cases and deaths,* by sex, racial/ethnic group,† and age group — National Program of Cancer Registries, and Surveillance, Epidemiology, and End Results Program, United States, 2011

	Incidence§								
	
	Overall			Men			Women		
			
Characteristic	No.	Rate	95% CI	No.	Rate	95% CI	No.	Rate	95% CI
**All races/ethnicities**	**65,647**	**19.7**	**19.5–19.9**	**38,415**	**25.3**	**25.1–25.6**	**27,232**	**15.6**	**15.5–15.8**
White	61,337	22.1	21.9–22.2	36,145	28.0	27.7–28.3	25,192	17.8	17.5–18.0
White, Hispanic	1,266	4.2	3.9–4.4	551	4.4	4.0–4.8	715	4.2	3.9–4.6
White, non-Hispanic	60,071	24.6	24.4–24.8	35,594	30.8	30.5–31.1	24,477	20.0	19.8–20.3
Black	359	1.0	0.9–1.2	153	1.1	0.9–1.3	206	1.0	0.9–1.2
Asian/Pacific Islander	197	1.3	1.1–1.5	87	1.4	1.1–1.7	110	1.3	1.1–1.6
American Indian/Alaska Native	128	4.3	3.6–5.2	72	6.2	4.6–8.0	56	3.2	2.4–4.2
Hispanic	1,371	4.1	3.9–4.4	590	4.3	3.9–4.7	781	4.2	3.9–4.5
**Age group (yrs)**									
<15	129	0.2	0.2–0.3	59	0.2	0.1–0.2	70	0.2	0.2–0.3
15–19	217	1.0	0.9–1.2	85	0.8	0.6–1.0	132	1.3	1.1–1.5
20–24	722	3.3	3.1–3.5	199	1.8	1.5–2.0	523	4.9	4.5–5.3
25–29	1,348	6.4	6.1–6.7	438	4.1	3.7–4.5	910	8.7	8.2–9.3
30–34	1,854	9.1	8.7–9.5	688	6.8	6.3–7.3	1,166	11.5	10.8–12.2
35–39	2,242	11.5	11.1–12.0	902	9.3	8.7–10.0	1,340	13.8	13.0–14.5
40–44	3,206	15.4	14.9–15.9	1,353	13.1	12.4–13.8	1,853	17.7	16.9–18.5
45–49	4,568	20.8	20.2–21.4	2,130	19.6	18.8–20.5	2,438	21.9	21.1–22.8
50–54	6,071	27.1	26.4–27.8	3,258	29.7	28.7–30.7	2,813	24.7	23.8–25.6
55–59	6,736	33.5	32.7–34.3	3,950	40.6	39.3–41.9	2,786	26.9	25.9–27.9
60–64	7,748	43.9	42.9–44.9	4,942	58.4	56.8–60.0	2,806	30.5	29.4–31.7
65–69	7,355	57.7	56.3–59.0	4,873	81.0	78.8–83.3	2,482	36.8	35.4–38.3
70–74	6,694	70.3	68.6–72.0	4,580	105.0	102.0–108.1	2,114	41.0	39.2–42.8
75–79	6,135	83.7	81.6–85.8	4,176	130.3	126.4–134.3	1,959	47.5	45.4–49.6
80–84	5,603	97.6	95.0–100.1	3,701	159.5	154.4–164.8	1,902	55.6	53.1–58.1
≥85	5,019	88.3	85.9–90.8	3,081	164.4	158.7–170.3	1,938	50.9	48.7–53.2

	**Mortality**								
	
	**Overall**			**Men**			**Women**		
			
**Characteristic**	**No.**	**Rate**	**95% CI**	**No.**	**Rate**	**95% CI**	**No.**	**Rate**	**95% CI**

**All races/ethnicities**	**9,128**	**2.7**	**2.6–2.7**	**6,001**	**4.0**	**3.9–4.1**	**3,127**	**1.7**	**1.6–1.7**
White	8,928	3.1	3.0–3.2	5,906	4.6	4.5–4.7	3,022	1.9	1.9–2.0
White, Hispanic	232	0.9	0.7–1.0	133	1.1	0.9–1.3	99	0.7	0.5–0.8
White, non-Hispanic	8,687	3.4	3.3–3.4	5,769	5.0	4.9–5.1	2,918	2.1	2.0–2.2
Black	133	0.4	0.3–0.5	56	0.4	0.3–0.5	77	0.4	0.3–0.5
Asian/Pacific Islander	46	0.3	0.2–0.4	23	0.4	0.2–0.6	23	0.3	0.2–0.4
American Indian/Alaska Native	21	0.8	0.5–1.2	16	1.3	0.7–2.2	—¶	—¶	—¶
Hispanic	235	0.8	0.7–0.9	134	1.0	0.8–1.2	101	0.6	0.5–0.8
**Age group (yrs)**									
<15	—¶	—¶	—¶	—¶	—¶	—¶	—¶	—¶	—¶
15–19	—¶	—¶	—¶	—¶	—¶	—¶	—¶	—¶	—¶
20–24	22	0.1	0.1–0.2	—¶	—¶	—¶	—¶	—¶	—¶
25–29	56	0.3	0.2–0.3	35	0.3	0.2–0.5	21	0.2	0.1–0.3
30–34	103	0.5	0.4–0.6	63	0.6	0.5–0.8	40	0.4	0.3–0.5
35–39	151	0.8	0.7–0.9	78	0.8	0.6–1.0	73	0.7	0.6–0.9
40–44	282	1.3	1.2–1.5	169	1.6	1.4–1.9	113	1.1	0.9–1.3
45–49	435	2.0	1.8–2.2	262	2.4	2.1–2.7	173	1.5	1.3–1.8
50–54	629	2.8	2.6–3.0	395	3.6	3.2–3.9	234	2.0	1.8–2.3
55–59	828	4.1	3.8–4.4	557	5.7	5.2–6.2	271	2.6	2.3–2.9
60–64	1,024	5.7	5.4–6.1	698	8.2	7.6–8.8	326	3.5	3.1–3.9
65–69	997	7.7	7.3–8.2	704	11.6	10.8–12.5	293	4.3	3.8–4.8
70–74	1,035	10.8	10.1–11.4	731	16.6	15.4–17.8	304	5.8	5.2–6.5
75–79	1,139	15.4	14.5–16.3	784	24.3	22.6–26.0	355	8.5	7.7–9.5
80–84	1,095	18.9	17.8–20.1	743	31.8	29.5–34.2	352	10.2	9.2–11.3
≥85	1,327	23.2	22.0–24.5	768	40.7	37.9–43.7	559	14.6	13.4–15.9

* Per 100,000 persons, age-adjusted to the 2000 U.S. standard population.

† Racial categories are not mutually exclusive from Hispanic ethnicity unless noted. Rates are not presented for cases with unknown or other race.

§ Compiled from cancer registries that meet the data-quality criteria for all invasive cancer sites combined (covering approximately 99% of the U.S. population).

¶ Value not displayed because there are fewer than 16 deaths.
